# Int Braz J Urol Annual Report – 2019 and new Editor

**DOI:** 10.1590/S1677-5538.IBJU.2019.01.01

**Published:** 2019

**Affiliations:** 1 Departamento de Urologia, Faculdade de Medicina do ABC, Santo André, SP, Brasil

This is the first issue of 2019, a special year for *International Brazilian Journal of Urology*. It will complete 45 years of uninterrupted publication of our magazine. This is a landmark, since Int Braz J Urol is a journal always supported by the Brazilian Society of Urology, without the aid of any other publishing company. Also, this is an open access journal that has never charged the authors for any publishing fee.

In the end of the current year, my second and last term as Editor in Chief will end, according to the Internal Regiment of SBU. We will begin the process of selecting a new Editor, who will edit Int Braz J Urol with me during the second semester and will assume the position in January, 2020.

It has been an honor and a pleasure to edit this Journal, along with an excellent group of Associated Editors, with renowned and participative Consulting Members, and a high quality technical group for support. In 2018, we received 762 papers from Brazil and several parts of the Word. Our 1,232 reviewers evaluated and accepted 169 articles. Many authors of good papers could not publish their work at Int Braz J Urol due to limited space, and we thank them all for choosing our Journal to publicize their research.

The reviewers deserve a special attention. They are the soul of any scientific journal, since it is a voluntary and most of the times anonymous job, almost exclusively performed in the name of the art of Science and scientific publication. To all of them, my sincere acknowledgment.

As in every year, Int Braz J Urol announces the most effective reviewers. Evaluation is based on the number of evaluated papers, number of accepted invitations for revision, time to review and quality of revision. In 2018, these are the six more effective reviewers: Victor Espinheira Santos, MD, (Núcleo de Urologia, Hospital da Bahia, Brazil); Sarica Kemal, MD, (Department of Urology, Kartal Dr. Lufi Kirdar Training and Research Hospital, Turkey); Giovani Marchini, MD, Divisão de Urologia, Hospital das Clínicas da Faculdade de Medicina da Universidade de São Paulo, SP, Brazil); Aley Talans, MD, (Departmento de Radiologia, Hospital das Clínicas da Faculdade de Medicina da Universidade de São Paulo, SP, Brazil); Victor Tonso, MD, (Departamento de Radiologia e Diagnóstico por Imagem, Hospital Israelita Albert Einstein, SP, Brazil). Trushar Patel, MD, (Department of Urology, University of South Florida) was elected the best reviewer of the videos section.

The former “Jornal Brasileiro de Urologia” was founded in 1974 as a scientific periodic were the Brazilian urologists could publish their work. Over the years, it became an International Journal, changed its name, was indexed in the main international database and gained the World, maintaining its Brazilian characteristics. I am sure that every Brazilian urologist must be proud of these 45 years!

